# Bilateral Stellate Nonhereditary Idiopathic Foveomacular Retinoschisis (SNIFR) Incidentally Identified in a Non-Myopic Female

**DOI:** 10.7759/cureus.42805

**Published:** 2023-08-01

**Authors:** Asli Perente, Doukas Dardabounis, Irfan Perente, Aristeidis Konstantinidis, Georgios Labiris

**Affiliations:** 1 Ophthalmology Department, University Hospital of Alexandroupolis, Alexandroupolis, GRC

**Keywords:** optical coherence tomography, retinal splitting, foveoschisis, congenital x-linked retinoschisis, stellate nonhereditary idiopathic foveomacular retinoschisis

## Abstract

Stellate foveomacular retinoschisis is commonly associated with congenital X-linked retinoschisis, which is almost exclusively seen bilaterally in males. In the absence of a family history of retinoschisis and other related conditions, the term stellate nonhereditary idiopathic foveomacular retinoschisis (SNIFR) is used. SNIFR constitutes a rather rare diagnosis and is usually observed unilaterally in myopic females. Within this context, we report a case of a non-myopic female patient with bilateral SNIFR detected with optical coherence tomography (OCT).

## Introduction

Foveomacular retinoschisis or foveoschisis is the term used to describe the separation of retinal layers involving the central macula [1]. Congenital X-linked retinoschisis constitutes a common reason for foveoschisis caused by the mutation in the RS1 gene. Other etiologies include enhanced S-cone syndrome, myopic traction maculopathy, optic disc pit maculopathy, glaucoma, vitreomacular traction, and niacin or taxane-induced foveoschisis [2, 3]. Stellate nonhereditary idiopathic foveomacular retinoschisis (SNIFR) is a new classification recently introduced by Ober MD et al. to describe cases with foveomacular retinoschisis without hereditary background or other predisposing conditions [4]. It typically appears in otherwise healthy asymptomatic individuals who retain visual acuity of 20/40 or better. Because of its benign course, most cases do not need any therapeutic intervention, while annual optical coherence tomography (OCT) monitoring is suggested [3]. According to the largest published case series of SNIFR, most of the patients were females, and most were myopic with unilateral disease [4]. We report herein a case of bilateral SNIFR incidentally identified in a non-myopic female.

## Case presentation

**Figure 1:**
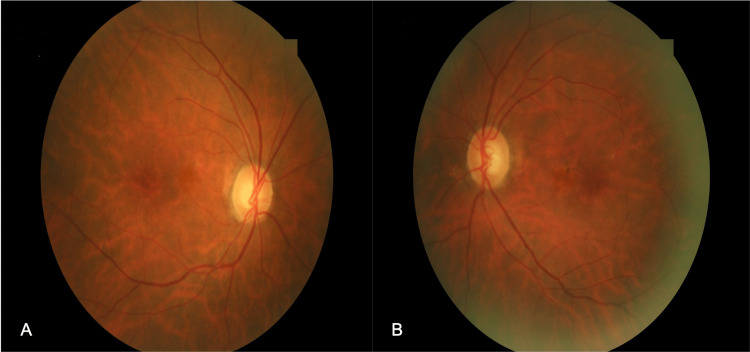
Color fundus photographs of the right (A) and the left (B) eye showing retinal pigment epithelium changes and yellowish deposits around the fovea.

**Figure 2:**
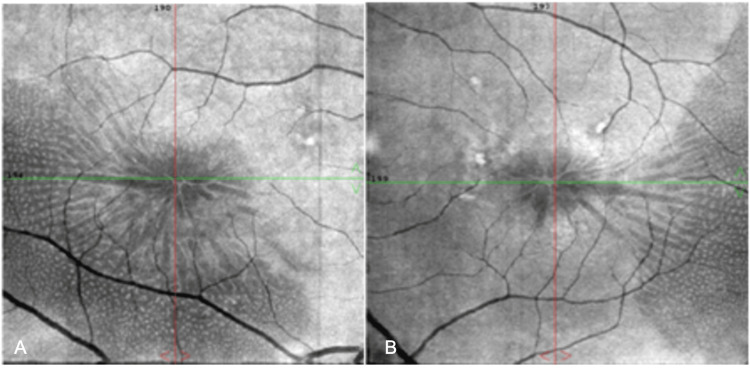
En-face OCT images of the right (A) and the left (B) eye with evident radial spoking. OCT: Optical coherence tomography.

**Figure 3:**
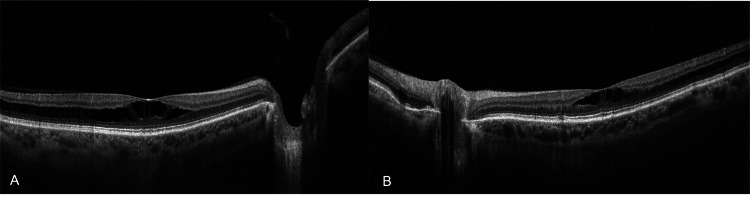
Optical coherence tomography (OCT) of the right (A) and the left (B) eye showing splitting of the retina.

**Figure 4:**
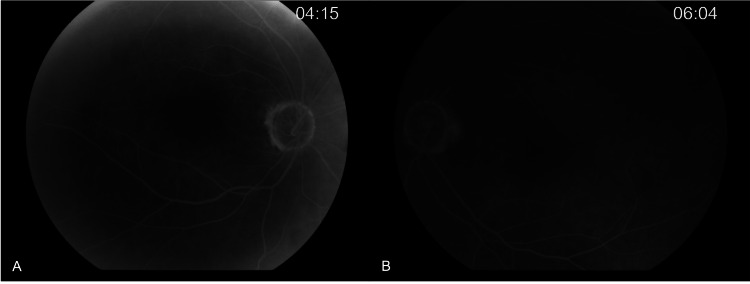
Late-phase fluorescein angiography (FA) of the right (A) and the left (B) eye showing normal retinal circulation.

A 74-year-old female presented to the outpatient service of our department complaining of bilateral progressive vision loss. Her previous medical, ocular, and family history was unremarkable. The best corrected visual acuity (BCVA) was 20/32 (Snellen) with +1.25 sphere in both her eyes (OU). The intraocular pressure (IOP) was 13 mmHg in the right eye (OD) and 15 mmHg in the left eye (OS). Furthermore, the slit lamp examination revealed cataracts with stage 2 nuclear opalescence according to the Lens Opacities Classification System III (LOCS-3) grading scale OU. On dilated fundus examination, the optic nerves of both eyes appeared with distinct margins and the estimated cup-to-disc ratio of 0.3 in OD and 0.4 in OS. At the same time, retinal pigment epithelium (RPE) changes with small yellowish deposits in the macula of both eyes were observed (Figure 1). En-face OCT of the outer retina revealed radial spoking around the fovea OU (Figure 2). The OCT detected splitting of the outer plexiform layer at the macula extending temporally OU without evident vitreomacular traction (Figure 3). The fundus fluorescein angiography (FA) did not detect any underlying condition (Figure 4). On optical biometry, the axial length of her OD was found to be 23.29 mm, and the OS had a length of 23.18 mm. The patient was diagnosed with bilateral SNIFR. Dorzolamide 2% four times a day was administered; however, no positive response was observed during three months of therapy. Nine months later, the patient had stable visual acuity and OCT findings, and she was scheduled for annual monitoring.

## Discussion

Retinoschisis, a clinical entity known since the late 19th century, was originally described by Jager in 1953. Although different etiologies can lead to retinoschisis involving the macular region, the underlying mechanism in all cases is thought to be the contraction of the posterior vitreous cortex or anomalies in protein synthesis [5, 6]. SNIFR is a newer classification of foveoschisis used to describe patients without a personal or familial history of juvenile X-linked retinoschisis and other risk factors, thus making it a diagnosis of exclusion [4]. Even though these two conditions share similar clinical findings, the location of the retinal splitting differs. Specifically, in juvenile X-linked retinoschisis, the inner nuclear layer is primarily affected, whereas in SNIFR, the splitting of the retina involves Henle’s fiber layer [7]. Fragiotta S et al. investigated these structural differences using swept-source OCT findings. According to their results, in the case of X-linked juvenile retinoschisis, OCT angiography (OCTA) showed vascular flow in the affected areas because of the presence of ‘’bridging’’ vessels within the inner nuclear layer. On the contrary, the OCTA of the patient with SNIFR did not visualize blood flow in the avascular Henle’s fiber layer [7]. Given the challenge of detecting these conditions through clinical examination alone, Montano M et al. emphasized the crucial role of OCT and OCTA in illustrating the novel findings of SNIFR [8]. In fact, in our case, the OCT set the suspicion of the diagnosis, indicating the role of multimodal imaging in routine clinical practice. According to the study of Ober MD et al., the most common refractive error seen in SNIFR was myopia, and 12 out of 22 of their patients had unilateral disease [4]. Panos GD et al. reported a case of a hyperopic female with unilateral SNIFR [2], while McBride M et al. described a male patient with unilateral hyperopic refraction and SNIFR [9]. The occurrence of SNIFR bilaterally in hyperopic eyes, like in our case, is considered a rarity and has been reported in the literature only once before [10]. When it comes to managing SNIFR, most patients maintain stable visual acuity and only require annual monitoring. Nonetheless, significant deterioration of visual acuity and the development of subretinal fluid indicate the need for therapeutic intervention [10]. While vitrectomy with tamponade and internal limiting membrane peeling is the therapeutic intervention of choice, more conservative options such as the administration of topical dorzolamide and intravitreal injection of bevacizumab with both controversial results have been reported in the literature [1, 3, 11, 12]. Ajlan RS et al. described a unique case of SNIFR treated successfully with topical dorzolamide hydrochloride 2% [13].

## Conclusions

In this paper, we describe a case of a hyperopic female patient with bilateral SNIFR, which was incidentally detected during a routine clinical examination. Genetic testing was not conducted, as the patient had no history of reduced visual acuity or night blindness from an early age. Niacin and taxane-induced foveoschisis were also ruled out based on her medical history. Furthermore, vitreomacular traction was not detected on the OCT, and the FA excluded the presence of any underlying vascular disease. Our report suggests that, though extremely rare, the detection of bilateral SNIFR in a non-myopic female is possible, and the use of multimodal imaging could be instrumental in such cases.
